# Early Evaluation of IMAGINATOR 2.0 Intervention Targeting Self-Harm in Young People: Single-Arm Feasibility Trial

**DOI:** 10.2196/79496

**Published:** 2026-01-26

**Authors:** Athina Servi, Emily Gardner-Bougaard, Saida Mohamed, Aaron McDermott, Rachel Rodrigues, Ben Aveyard, Nejra Van Zalk, Adam Hampshire, Lindsay Dewa, Martina Di Simplicio

**Affiliations:** 1 Department of Brain Sciences Faculty of Medicine Imperial College London London United Kingdom; 2 West London NHS Trust London United Kingdom; 3 Dyson School of Design Engineering Imperial College London London United Kingdom; 4 Institute of Psychiatry, Psychology & Neuroscience King's College London London United Kingdom; 5 School of Public Health Imperial College London London United Kingdom; 6 NIHR Imperial Biomedical Research Centre London, England United Kingdom

**Keywords:** self-harm, intervention, digital, mental imagery, young people, adolescents, co-production

## Abstract

**Background:**

Self-harm (SH) affects around 20% of all young people in the United Kingdom. Treatment options for SH remain limited and those available are long and costly and may not suit all young people. There is an urgent need to develop new scalable interventions to address this gap. IMAGINATOR is a novel imagery-based intervention targeting SH initially developed for individuals aged 16 to 25 years. It is a blended digital intervention delivering functional imagery training via therapy sessions and a smartphone app.

**Objective:**

This study aimed to pilot a new version of the app, IMAGINATOR 2.0, extended to adolescents from the age of 12 years and coproduced with a diverse group of young people with lived experience. Our aim was also to test the feasibility and acceptability of delivering IMAGINATOR 2.0 in secondary mental health services.

**Methods:**

A total of 4 co-design workshops were conducted online with UK-based lived-experience co-designers aged 14-25 years to develop the IMAGINATOR 2.0 app. The intervention was then piloted with participants recruited from West London NHS Trust Tier 2 Child and Adolescent Mental Health Services and adult Mental Health Integrated Network Teams. Participants received 3 face-to-face functional imagery training sessions in which the app was introduced and 5 brief phone support sessions. Outcome assessments were conducted after completing therapy, approximately 3 months post baseline. Two focus groups gathered the therapists’ perspectives on IMAGINATOR 2.0’s acceptability and means of improvement. For quantitative data, descriptives are reported. Qualitative data were analyzed using a coproduced thematic analysis method with young people with lived experiences.

**Results:**

Overall, 83 participants were referred, and 29 (gender: n=28 women, n=1 transgender; mean age 18.9, SD 3.74 years) were eligible and completed screening. Of the 27 participants who started, 59% (n=16) completed therapy per protocol, while only 15 (55.6%) completed the quantitative outcome assessment. There was an overall reduction in the number of SH episodes over 3 months from pre- to postintervention (baseline: median 7, IQR 3.5-21.5 months; postintervention: median 0, IQR 0-7 months; median difference=–6.5; *r*=0.69). Six themes were identified through thematic analysis of therapists’ feedback, including mental imagery’s potential and boundaries, therapy expectations, experience and effectiveness, accessibility of digital support, and adaptation of the IMAGINATOR 2.0 app to complement care pathways. The app was valued by therapists who highlighted the need for an intervention like IMAGINATOR 2.0 in their services.

**Conclusions:**

IMAGINATOR 2.0 shows initial promise as an acceptable brief intervention targeting SH in young people under adolescent and adult mental health services. Challenges with attrition need to be addressed for a definitive randomized controlled trial to test the intervention efficacy.

**Trial Registration:**

ClinicalTrials.gov NCT06311084; https://clinicaltrials.gov/study/NCT06311084

## Introduction

Self-harm (SH) is defined as the “intentional act of self-poisoning or self-injury, irrespective of the motivation” [1]. It is an expression of emotional distress that typically begins in early adolescence and peaks in frequency around the age of 16 years [2]. SH prevalence has increased over the past 20 years [2], with approximately 20% of individuals aged 16-25 years reporting having self-harmed at some point in their life [2-4]. SH is associated with poor health and functional outcomes, including risk of suicide [5]. Despite the impact on young people’s functioning, available targeted interventions are inadequate to address the high level of need [6,7]. Interventions with the strongest evidence support (such as cognitive behavioral therapy [CBT] [8] or dialectic behavioral therapy [9]) are either limited to adults or have long duration and high cost and require a high level of commitment. Therefore, we lack a stepped care model for SH [10], starting with a brief early intervention for SH in young people prior to long, complex treatments. Digital solutions could be crucial to bridge this gap and show promise in reducing the high prevalence of SH [11] by providing early support to large numbers of young people [12]. Young people are open to digital interventions for SH, especially if they provide coping strategies, but not as a substitute for human support [13]. However, despite their acceptability, challenges such as reduced app usage over time have been noted [14], while only a handful of app or web-based interventions for SH focus on and were co-designed with young people and have been adequately tested [15-17].

SH cognitions are often experienced in the form of mental imagery: vivid, realistic mental representations that can entail re- or pre-experiencing the same emotional and physiological reactions as the actual act [18]. SH mental imagery has been identified in clinical and student populations (84% and 73% prevalence, respectively) [19,20] and has been shown to increase the urge and likelihood of future SH [21-23]. Nevertheless, mental imagery of adaptive actions can promote engagement in adaptive behavior [24]. Interventions including mental imagery techniques, either transforming unhelpful mental images or promoting helpful ones, have shown growing potential in conditions related to SH, including depression [25], emotion dysregulation [26], suicidal ideation in students [27], and SH itself in adolescents [26].

Studies show that mental imagery interventions such as functional imagery training (FIT) can be more effective than motivational interviewing at promoting change of problematic behaviors [28-30]. FIT [31] involves training and rehearsal of motivational imagery to enhance the desire to reach adaptive goals [32]. For example, it has been shown to promote weight loss [30] and reduce alcohol consumption [29], suggesting it could help individuals in identifying and implementing alternative strategies to replace SH. IMAGINATOR is an imagery-based intervention delivering FIT to target SH via a combination of face-to-face therapy sessions and a smartphone app. The proposed therapeutic ingredient is personalized, positive, future-oriented imagery to support behavioral control. During FIT, individuals develop a personal imagery plan (like a movie to play in one’s mind) of an adaptive behavior to put in place when and where the urge to SH occurs. In a proof-of-concept study, we showed that IMAGINATOR reduced SH after 3 months, which was maintained at 6 months in young people aged 16-25 years [33].

Building on these results, we co-designed a new version of the app for adolescents and young adults aged 12-25 years (IMAGINATOR 2.0). In this paper, we will refer to this age group as “young people.” Our study aimed to assess the feasibility, acceptability, and safety of IMAGINATOR 2.0, in particular, piloting the use of the new app and the intervention delivery in children, adolescents, and adult secondary mental health services. Secondary objectives were to examine change in key outcomes, such as the number of SH episodes, SH urges’ intensity and SH mental imagery characteristics, other mental health symptomatology, and explore clinicians’ perspectives in delivering IMAGINATOR 2.0. Examining changes in SH outcomes provides preliminary insight into potential intervention effects to inform future trials. Young people’s views are reported in a separate qualitative paper [34].

## Methods

### Design

This was a single-arm study delivering FIT as a low-intensity intervention alongside standard care in a sequential sample of young people under the care of West London NHS Trust. The study was registered with ClinicalTrials.gov (NCT06311084) prior to participant enrollment.

### Patient and Public Involvement

A Young Person Advisory Group of 4 young people with lived experience of SH was involved in the funding application, design, recruitment, data collection, data analysis, interpretation of results, and dissemination. Details on the group’s involvement are reported elsewhere [34]. Three members (SM, AM, and NC) took part in the final stages of the thematic analysis reported here. They revised codes and generated themes together with the rest of the research team in 2 online meetings in April 2024.

### Co-Design of the IMAGINATOR 2.0 App

**Figure 1:**
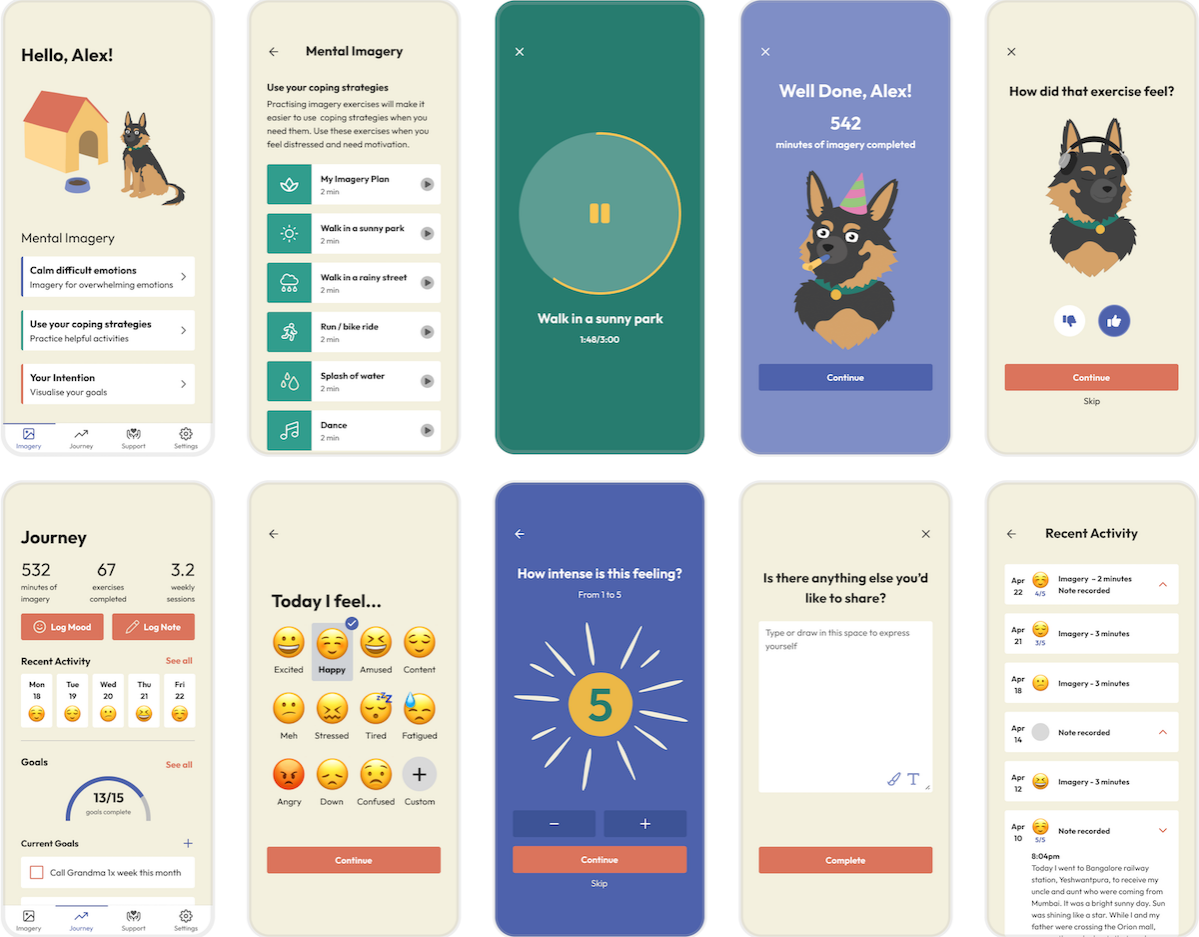
Main pages and appearance of the IMAGINATOR 2.0 app. The top panel shows the main imagery dashboard with all imagery audios for young people to practice (top left) and the user journey to selecting a subgroup of imagery audios (“coping strategies”), a specific audio to listen to (“walk in sunny park”), the mascot support, and giving feedback on the imagery exercise. The bottom panel illustrates the Journey tab, where the young people can track mood, notes, and goals, and the mood rating functionality: from selecting an emoji, to rating the feeling intensity, adding notes, and reviewing activities and mood. Additional tabs included Support, with crisis support resources, where young people can also add personal phones from people in their contacts, and Settings, where young people have the option to enable reminders for practicing mental imagery and logging mood.

We co-designed a new app with a diverse group of 14 young people with lived experience of SH recruited via social media across the United Kingdom. Details of the co-design process are reported elsewhere (case study [35] and Rodrigues et al., in preparation). Key functionalities selected for the final app (shown in Figure 1) included a degree of personalization, segmentation, inclusivity, and modularity (eg, a mascot to facilitate engagement, a goals tracker, a mood tracker, and a journal).

### Participants and Recruitment

Participants were recruited from Child and Adolescent Mental Health Services Tier 2 services and Mental Health Integrated Network Teams (adult community) in West London NHS Trust. Potential participants were identified and initially approached by their treating clinicians. Participants were eligible if they had experienced at least 2 SH episodes (an occurrence of SH perceived as distinct in time, precipitants, motives, initiation, and conclusion) in their life with at least 1 SH episode in the past month, or at least 5 SH episodes in the past year and currently reporting SH urges (self-report of wanting to engage in SH); had a smartphone; were fluent in English; and were willing to provide informed consent. Participants were excluded if they had a severe learning disability or pervasive developmental disorder, a current acute psychotic episode, current substance dependence, high risk of suicide or harm to others (based on clinicians’ risk assessment), or were taking part in concurrent treatment studies for SH or in concurrent psychological therapy.

### Procedures

Recruitment took place over 9 months from November 2022 to September 2023. Potential participants completed a baseline screening either in person or via Microsoft Teams video call (depending on participant preference), including assent or consent taking, sociodemographic characteristics (age, gender, ethnicity, sexual orientation, education, employment, and socioeconomic status), and the measures listed below using Qualtrics online surveys.

### Intervention

**Figure 2:**
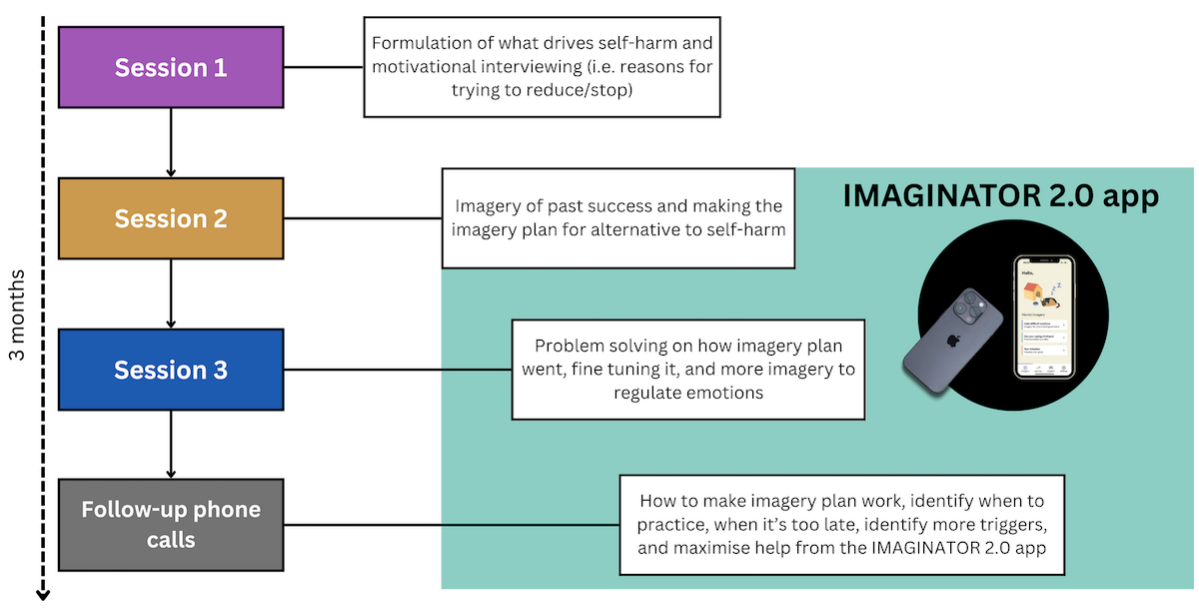
Overview of IMAGINATOR 2.0 intervention FIT sessions.

Eligible participants were assigned a therapist from the referring team (clinical psychologists, children and well-being practitioners, clinical assistant psychology therapists, or graduate mental health workers). These are psychology or mental health graduates with varying levels of postgraduate training (from none, graduate mental health workers, to 1-year CBT training, children and well-being practitioners, to 2 years’ Master’s-level training, clinical assistant psychology therapists, to clinical doctorate for the clinical psychologist). IMAGINATOR 2.0 consisted of the co-designed IMAGINATOR 2.0 app, three 1-hour FIT sessions in person or via Microsoft Teams once a week, and five 15- to 30-minute support phone calls fortnightly delivered after the three 1-hour FIT sessions. FIT comprised three main sessions: (1) session 1 completes an individualized formulation of personal drivers of SH behavior and a motivational interview on SH reduction combined with mental imagery techniques. (2) Session 2 starts with a mental imagery exercise of a past achievement to increase self-efficacy. This is followed by setting personal smart goals that include both directly targeting SH (eg, engaging in a desired behavior incompatible with or alternative to SH) and targeting other processes or symptoms that have been linked to SH in the formulation (eg, engaging in a behavior that reduces anxiety or rumination). Finally, a functional imagery plan is developed to achieve the goals. (3) Session 3 practices and refines the functional imagery and problem solves any challenges. The IMAGINATOR 2.0 app was introduced in session 2. The app’s key feature is mental imagery audios to support practicing the functional imagery plan at home, alongside affect and goal-tracking functions. Therapists encourage exploring which audios to use when tailored to individual needs (eg, different audios for different affect states). Follow-up phone calls focused on refining functional imagery practice, problem-solving, motivational encouragement, and personalization of the app use (Figure 2). The therapist also conducted a risk assessment at the beginning of each session or call.

Therapists were trained in the delivery of IMAGINATOR 2.0 over 2 workshops (1.5 days in total), provided with a manual, and supported via weekly group supervision over Microsoft Teams with the study principal investigator (MDS) and the research assistant (AS). During supervision, therapists illustrated individual cases and were guided by MDS to maintain adherence to the treatment protocol. Therapists also discussed strategies to address emerging challenges, including how to adapt and tailor FIT around each individual’s needs and maintain engagement with the app.

### Outcome Assessments

Participants completed the outcome assessment after the final phone session (approximately 3 months from baseline) via Microsoft Teams video call and followed the same procedures and measures as the baseline assessment. All were then invited to further expand on their answers in a semistructured interview about their experience using IMAGINATOR 2.0 [34].

### Focus Groups

Given the absence of previous qualitative studies on using a digital imagery–based intervention for SH and the heterogeneity of therapists (different training and service lines), we chose a focus group format so that therapists could share and reflect on their experiences and be exposed to different viewpoints, facilitating a broader exploration of themes and depth of discussion. Therapists were invited to discuss their experience in delivering IMAGINATOR 2.0 across 2 parallel focus groups that took place in person in a private room on December 8, 2023, and December 13, 2023, respectively, on a university campus. They took approximately 2 hours and were cofacilitated by the principal investigator (MDS) and 2 other researchers (AS and EGB). A semistructured interview guide with open-ended questions was coproduced with the Young Person Advisory Group and used to guide discussions (Multimedia Appendix 1). Topics included barriers to adopting the intervention, perceived benefits, and suggestions for improvement. The sessions were audio-recorded with therapists’ consent, transcribed verbatim, and anonymized. Field notes were also taken by the assisting researchers (AS and EGB) to capture nonverbal cues and group dynamics.

### Measures

The primary outcome was feasibility and acceptability. Feasibility was measured by attrition rate (percentage of enrolled eligible participants completing outcome assessment) and treatment adherence (percentage of participants completing intervention per protocol, ie, 5/8 sessions). Acceptability was captured using the Client Satisfaction Questionnaire [36] and the User Experience Questionnaire [37] (Multimedia Appendix 2). Adverse events, defined as any untoward medical occurrence in a participant, were recorded at each assessment and therapy session. Serious adverse events were immediately reported by research assistants or therapists to the principal investigator (MDS), who discussed relatedness to the study procedures with the relevant clinical team.

Secondary outcomes included change in SH frequency and severity over the past 3 months, assessed as per the following measures:

1. SH frequency over the past 3 months: SH frequency was measured via the Timeline Follow-Back interview [33,38]. In this interview, the researcher reconstructs together with the participant the number of discrete SH episodes using calendar cues. The scale records the total number of SH episodes within a specified timeframe. The minimum number of episodes is 0, and the maximum number of episodes that can be reported for the 3 months is every day (around 90, depending on the number of days of the month). It has been successfully used in previous research measuring behavioral outcomes, such as in addiction studies and our proof-of-concept study [33]. We selected the Timeline Follow-Back technique, as the calendar-cued method aids better precision in recollecting SH retrospectively than a simple self-report questionnaire.
2. SH severity over the past 3 months: SH severity was measured using a Visual Analog Scale, severity criteria used in the nonsuicidal self-injury disorder in the *DSM-5 (Diagnostic and Statistical Manual of Mental Disorders* [Fifth Edition]) [39] and the number of different SH methods used (also termed SH versatility), elicited via these open questions: “In the last three months, how severe was the worst injury that you inflicted to yourself? Can you list all the ways that you have used to harm yourself in the last three months?” This method was advised by the Young Person Advisory Group to avoid scales listing numerous SH methods, which could be triggering or enabling.

Other secondary outcomes were the (1) Self-Harm Imagery Interview adapted from Di Simplicio et al [33] and Hales et al [40], the State Motivation for Reducing Self-Harm scale [41], the Craving Experience Questionnaire for Self-Harm, adapted from Craving Experience Questionnaire [42], the Revised Children’s Anxiety and Depression Scale [43] for participants aged 12-17 years and via the Depression, Anxiety and Stress Scale [44] for participants aged 18-25 years, the Warwick-Edinburgh Mental Well-being Scale [45], the Difficulties in Emotion Regulation Scale-Short Form [39], the 11-item behavior supplement to the Borderline Symptom List [46], the Alcohol Use Disorders Identification Test [47], and the Cannabis Use Disorder Identification Test Revised [48]. Rationale and description of full scales are reported in Multimedia Appendix 2.

### Analysis

#### Quantitative Data

Given the feasibility nature of the study, all quantitative data are reported as descriptive statistics with median values and IQRs, as data were not normally distributed. Effect sizes were calculated as r=z/N [49]. The interpretation of effect sizes was guided by Cohen *d* rule of thumb, where 0.2 denotes small, 0.5 medium, and 0.8 a large effect size [50].

Attrition, adherence to therapy per protocol, and number of therapy sessions completed are reported as counts and percentages. To explore the relationship between adherence to therapy and various demographic variables, we conducted a Mann-Whitney *U* test comparing the average number of therapy sessions completed between age, ethnicity, and sexual orientation groups.

Due to the small sample size, missing data were tolerated, and we assumed that the data were missing completely at random. Pairwise deletion was used to handle missing data. Out of 15 participants who completed the outcome assessment, data were missing from 1 participant over 5 measures (11-item behavior supplement to the Borderline Symptom List, Revised Children’s Anxiety and Depression Scale, Alcohol Use Disorders Identification Test, Cannabis Use Disorder Identification Test, and Difficulties in Emotion Regulation Scale-Short Form), 1 participant in 1 measure (State Motivation for Reducing Self-Harm scale), and 1 participant in another measure (Warwick-Edinburgh Mental Well-being Scale), due to surveys not being correctly saved on Qualtrics.

#### Qualitative Data

Qualitative data from the focus groups were analyzed using a coproduced reflexive thematic analysis approach guided by Braun and Clarke [51] and Dewa et al [52]. More details on reflexivity are reported in Multimedia Appendix 2. First, 2 researchers (AS and EG) familiarized themselves with the transcripts by reading and rereading them. Then, these 2 researchers coded the transcripts line by line independently. A Trello board was used to collate the initial codes separately added by both researchers, so that all team members could review these. Codes were then incorporated into a coding framework. The research team (MDS, LD, AS, and EG-B) and the coresearchers (SM, AMD, and NC) met twice online in April 2024 to review, refine, and finalize codes. We grouped codes based on shared meanings and continuously combined them until subthemes were formed with no repetitions. We then assigned provisional theme names and connected them to the subthemes. Once all themes and subthemes were finalized, we transferred them into Miro (RealtimeBoard, Inc) and reviewed them in isolation until we formed one final thematic map.

### Ethical Considerations

The study was approved by the West of Scotland Research Ethics Committee (Research Ethics Committee reference number 22/WS/0087) and the Health Research Authority. In accordance with UK legislation, participants aged 16 years and older provided informed consent prior to participation after a full explanation was given. For those aged 12-15 years, assent was obtained alongside parental or guardian consent. All participants were free to withdraw from the study at any point without giving reasons. All data processing procedures were in compliance with the UK Data Protection Act 2018. Data were pseudonymized. Participants received GBP £10 (US $13.49) per hour reimbursement in electronic vouchers for the outcome assessments and feedback interview (GBP £30 [US $40.48] overall).

## Results

### Sample

Sample demographics are reported in Table 1. Clinical characteristics are reported in Tables S1 and S2 in Multimedia Appendix 3. Among all participants (N=29), 21 (72.41%) reported experiencing suicidal ideation in the past month (assessed on the Columbia-Suicide Severity Rating Scale [53]) with 15 (51.72%) reporting active suicidal thoughts, 3 (10.34%) a specific plan and intent to act on the plan, and 3 (10.34%) suicidal attempts in the past month. Two participants had missing data on the Columbia-Suicide Severity Rating Scale during baseline.

**Table 1:** Demographic characteristics of eligible participants at baseline assessment (N=29).

Characteristics			Values
Age (years), mean (SD^a^)			18.86 (3.74)
**Gender, n (%)**			
	Woman	28 (96.55)	
	Transgender	1 (3.45)	
**Sexual orientation, n (%)**			
	Heterosexual	12 (41.38)	
	Other	12 (41.38)	
	LGBTQ+^a^	3 (10.34)	
	Not sure	2 (6.90)	
**Ethnicity, n (%)**			
	White	17 (58.62)	
	Black, African, Caribbean, or Black British	4 (13.79)	
	Mixed or multiple ethnic groups	3 (10.34)	
	Other ethnic group	3 (10.34)	
	Asian of Asian British	2 (6.90)	
**Household income (GBP^b^), n (%)**			
	<50,000	18 (62.07)	
	50,000-100,000	9 (31.03)	
	100,000-150,000	1 (3.45)	
	150,000-200,000	1 (3.45)	
**Highest level of education completed, n (%)**			
	Secondary education	13 (44.83)	
	Primary education	8 (27.59)	
	Higher education	7 (24.14)	
	Postgraduate education	1 (3.45)	
**Currently in education training, n (%)**			
	No	15 (51.72)	
	Yes	14 (48.28)	
**Currently employed, n (%)**			
	No	15 (51.72)	
	Yes	14 (48.28)	

^a^LGBTQ+: lesbian, gay, bisexual, transgender, queer or questioning, and other sexual and gender minority persons.

^b^1 GBP=US $1.35.

### Feasibility and Acceptability

**Figure 3:**
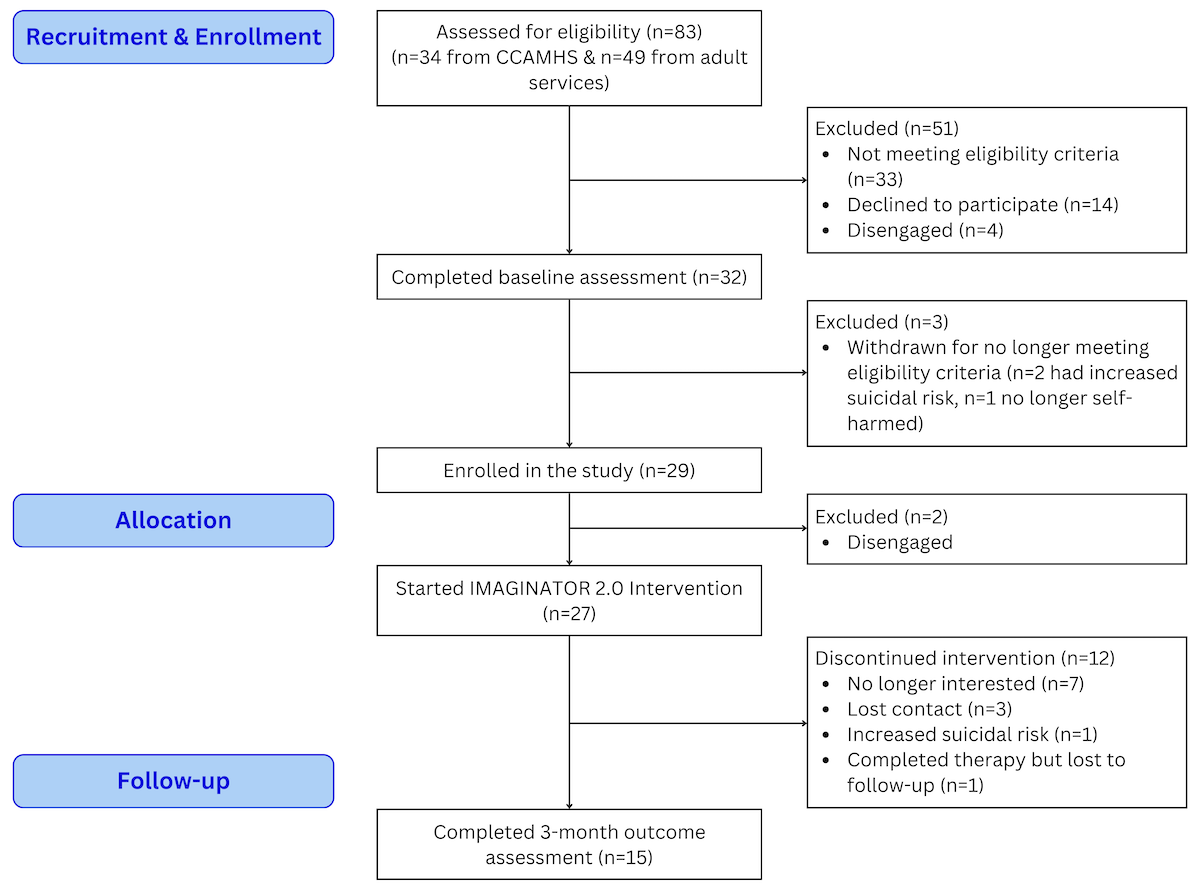
Flow diagram of participants in the IMAGINATOR 2.0 study. CAMHS: Child and Adolescent Mental Health Services.

Overall, 83 patients were referred to the study, of which 29 (34.94%) were both eligible and completed baseline screening (Figure 3). Out of these 29 patients, 27 (93.10%) started therapy. Out of the 27 who started therapy, 16 (59.3%) adhered to therapy per-protocol (all face-to-face sessions and at least 2 phone calls), 19 (70%) completed all face-to-face therapy sessions, and 9 (33.3%) completed all 5 follow-up phone calls. On average, the waiting time to start the intervention (see “Allocation”; Figure 3) was 4 (SD 2; range 0.7-9) weeks, while the average intervention duration was 21.3 (SD 6.9; range 9.7-36) weeks. Out of the 27 participants who started therapy, only 15 (55.6%) completed the follow-up outcome assessment.

The average number of therapy sessions completed out of the 3 face-to-face sessions and 5 follow-up phone calls (8 sessions total) was 4.93 (SD 2.80) and it significantly differed by sexual orientation with lesbian, gay, bisexual, transgender, queer or questioning, and other sexual and gender minority participants completing a higher number of sessions compared to heterosexual participants (Mann-Whitney *U* test=39.5; *P*=.01). No other demographic variable such as age or ethnicity differentiated therapy adherence.

Participants reported a median score of 25 (IQR 22-27.5) on the Client Satisfaction Questionnaire, indicating a very good satisfaction with the intervention received. Ratings on the User Experience Questionnaire referring to the app indicated good levels of perspicuity (median 1, IQR 0.5-2) and dependency (median 1.25, IQR 0.38-2), acceptable levels of attractiveness (median 1, IQR 0.25-1.83), but low levels of efficiency (median 1.25, IQR –0.13 to 1.5), stimulation (median 0.75, IQR –0.38 to 1.13), and novelty (median 0, IQR –0.5 to 1.13).

### Secondary Outcomes

**Figure 4:**
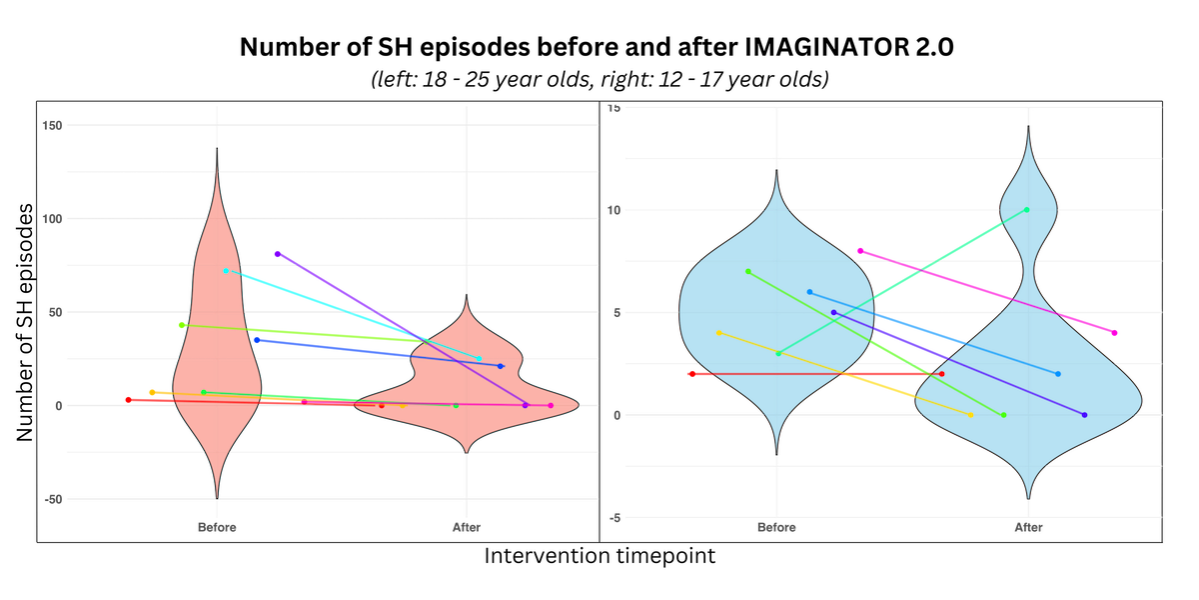
Number of SH episodes pre- and postintervention in 15 participants who completed both baseline and outcome assessments, grouped by age into Child and Adolescent Mental Health Services (<18 years old) and Mental health Integrated Network Teams (≥18 years old). Each participant was color-coded.

SH episodes reduced from a median of 7 (IQR 3.5-21.5) over the past 3 months at baseline to a median of 0 (IQR 0-7) over the past 3 months after IMAGINATOR 2.0 across all participants (effect size [*r*] 0.69, medium). Figure 4 shows participants categorized by age group.

Changes in clinical secondary outcomes are reported in Tables S1 and S2 (Multimedia Appendix 3), showing a reduction in craving for SH and an increase in participants’ motivation to reduce SH. Depression, anxiety, and stress also reduced in participants aged 18 years or older, while well-being and emotion regulation skills scores increased. Two participants reported increased suicidal risk between the baseline assessment and the start of the therapy sessions and were referred to a more intensive support team and consequently withdrew from the study. There were 98 adverse events during the study duration, corresponding to the number of SH episodes reported from baseline to the 3-month outcome assessment. One participant reported an increase in the number of SH episodes from baseline to outcome assessment (Figure 4). There were no serious adverse events, and no other adverse events were reported during the study.

### Qualitative Analysis From Focus Groups

#### Overview

**Figure 5:**
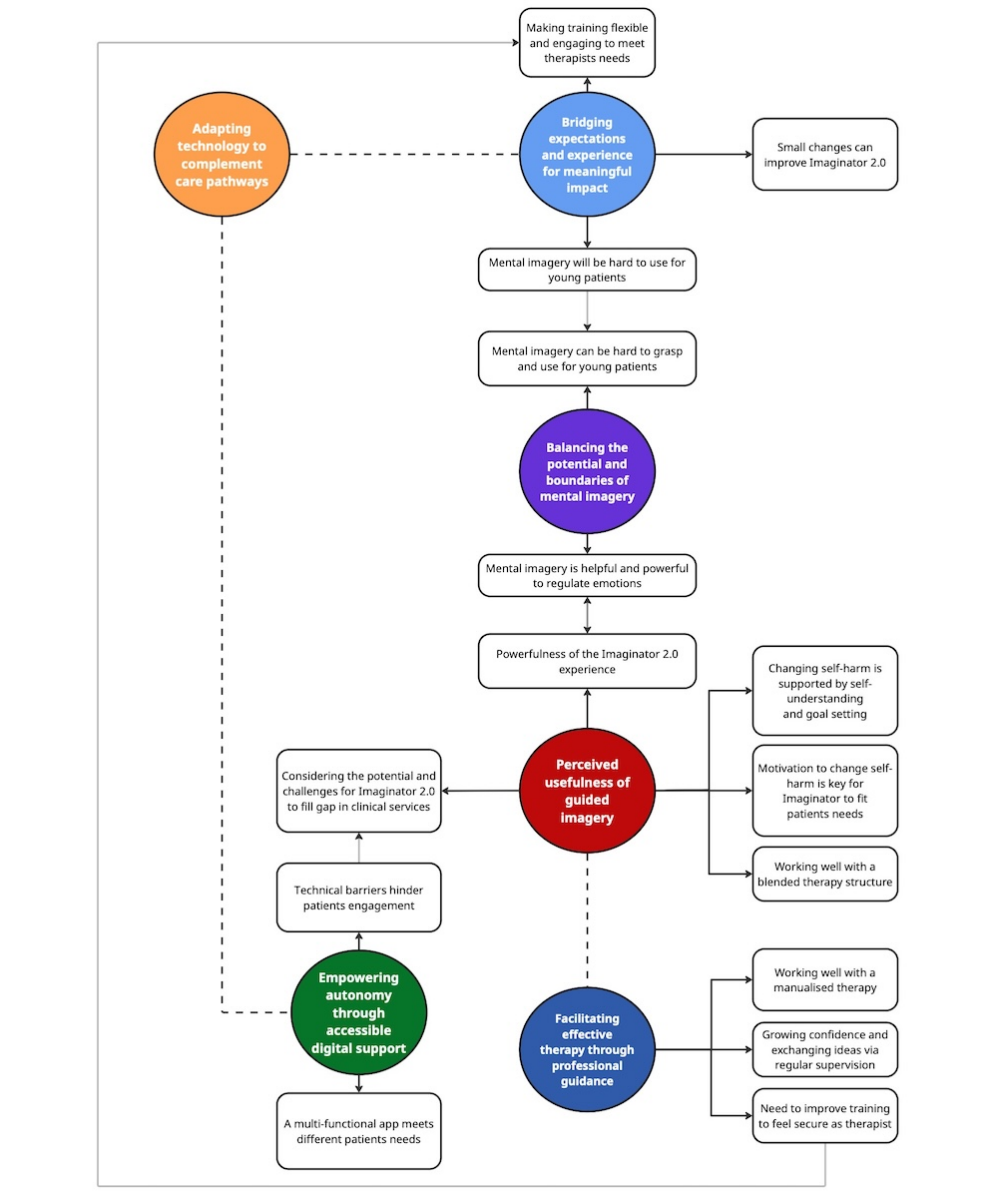
Coproduced thematic map of therapist focus group feedback. Solid arrows indicate the relations between each main theme and its corresponding subthemes, dashed lines represent conceptual links between main themes, and dotted arrows illustrate connections between subthemes across themes.

All 8 therapists took part in feedback focus groups: 2 clinical assistant psychology therapists, 2 graduate mental health workers, 1 assistant psychologist from adults services, 2 children and well-being practitioners, and 1 clinical psychologist from adolescent services. Thematic analysis identified six themes: (1) shifting self-perception through guided mental imagery, (2) empowering autonomy through accessible digital support, (3) balancing the potential and boundaries of mental imagery, (4) facilitating effective therapy through professional guidance, (5) adapting technology to complement care pathways, and (6) bridging expectations and experience for meaningful impact. Connections between themes and subthemes are conceptualized in a coproduced thematic map (Figure 5).

#### Perceived Usefulness of Guided Imagery

All 8 therapists reported that IMAGINATOR 2.0 was a powerful experience for patients that gave them an opportunity to understand SH beyond just risk assessments. For example, many therapists reported that it filled a critical gap in service provision, offering a needed short-term intervention targeting SH for young people that could reduce waiting times for this group. Many therapists commented that IMAGINATOR 2.0 equipped young people with coping skills to manage their SH and prevent escalation, and this could also help while waiting for more intensive therapy. Therapists also expressed that, preferably, there should not be a wait for IMAGINATOR 2.0 itself.

I think IMAGINATOR 2.0 was a really brilliant experience [..] Having [a] kind of a short-term therapy to reduce self-harm has been really helpful for my participants and they said they found it really useful.

Therapist ID: 6

Self-harm can escalate even though people are involved with [mental health services]. So, participants need something to just sort of contain the self-harm and IMAGINATOR 2.0 can do that quite well.

Therapist ID: 1

Most therapists agreed that patients referred to IMAGINATOR 2.0 should have the primary goal to change their SH behavior, or they may disengage from the intervention. Some felt that offering IMAGINATOR 2.0 to young people older than 14 years is preferable to younger children to make the concept of mental imagery easier to grasp. Others suggested that a longer history of SH could facilitate self-awareness and engagement. Understanding SH and goal setting were viewed as the key components of the therapy by almost all therapists.

I think understanding the self-harm and goal setting [are key components of the therapy] because that is what is going on and for them that was a big problem. Understanding what would be helpful for them.

Therapist ID: 3

If the self-harm was not such a big issue for them, they may not be as engaged and benefit from it as much, I guess.

Therapist ID: 5

Most therapists found the blended therapy approach (in-person, app, and calls) a good structure catering for patients’ different needs. However, some therapists reported patients were often distracted during phone calls and these sessions were not as efficient as face-to-face.

Overall, I sort of like the structure. I think it's sort of slowing down and basically having a repeated structure towards the end makes sort of the ending easier.

Therapist ID: 2

It’s just chaotic because you can hear in the background, they are playing video games or they're doing something else […] So I think that's a normal experience of phone calls with young people. And I think that's probably not as helpful for IMAGINATOR 2.0.

Therapist ID: 1

#### Balancing the Potential and Boundaries of Mental Imagery

All therapists highlighted that mental imagery was a powerful tool to regulate emotions and target the personalized goals set by young people. This was despite some young people finding it difficult to grasp and practice, and it had a mixed impact on SH for young people. Other key strengths of mental imagery reported across therapists included having the freedom to practice it anywhere at any time, the collaborative process of trial-and-error with the patient to identify the most helpful adaptive image, and the likelihood of them adopting mental imagery techniques in their future routine sessions with patients. A few therapists noted that mental imagery could elicit negative emotions, which occasionally led to other distressing or negative imagery.

One young person’s goals had more to do with social anxiety. They seemed to find it very useful to picture themselves in situations that they expected to find challenging and to actually vividly imagine themselves coping and having worked out what they would do in these situations. They found that helpful. It was a very collaborative process.

Therapist ID: 8

One of my participants sometimes found it hard to kind of stay on the image that she wanted. And her mind started to wander. And it started to go to negative places.

Therapist ID: 7

So even though they felt like mental imagery wasn't something that they like […], I think it just highlighted to them that if they set a goal, they can actually solve it, you know, work towards that goal, which I think was really helpful as well.

Therapist ID: 4

#### Bridging Expectations and Experience for Meaningful Impact

Most therapists expressed initial hesitation about using mental imagery as a tool to reduce SH in their patients. There was also some skepticism around how best to explain mental imagery and how it would work, and concern about the young age of patients being a barrier to understanding and using mental imagery techniques. For most, these negative expectations had dropped by the end of the intervention based on young people’s positive response to using imagery.

At the beginning, I was slightly sceptical, and I didn’t understand everything about the research. I was just wondering how helpful it could be and thinking in terms of imagery and how it would work. But then, after working with my first participant and recognising how she enjoyed it and how it was really working for her, I kind of changed the opinion I had initially.

Therapist ID: 7

So, the idea of using imagery seemed quite a compelling approach but I did find it quite challenging to deliver. Which I think was connected with the age group that I was working with.

Therapist ID: 8

Some therapists highlighted the need to focus on regulating emotions more directly, in addition to indirect approaches, to deal with SH urges. One therapist suggested integrating IMAGINATOR 2.0 as part of routine safety planning and training more people from the team to deliver it as an SH relapse prevention measure.

Using mental imagery as more than just an ad hoc tool for managing self-harm during my therapy sessions, has actually been quite effective with young people [outside IMAGINATOR 2.0]. When doing our routine safety plan, we discuss how they can use imagery. This could be part of our toolkit that we use in safety planning maybe.

Therapist ID: 1

Maybe first they do the managing emotions group to learn how to deal with difficult emotions and then if self-harm is still an issue put them on IMAGINATOR 2.0.

Therapist ID: 5

#### Facilitating Effective Therapy Through Professional Guidance

All therapists found the manual practical and a useful tool to prepare for sessions. They also found regular supervision sessions helpful, a safe space, and a learning session via hearing other people’s experiences. When faced with a challenge, supervision allowed for identifying alternative routes to approach a patient.

It was nice to have that sort of side [the manual] and the sense of knowing what direction to take to session. Sometimes with my client I found that we followed the manual a little bit but it's flexible in the sense that we don't have to sort of stick to the manual exactly. So, it’s quite helpful to have it there.

Therapist ID: 5

Most therapists found the 1-day workshop training informative, but were left with uncertainty around what the actual therapy was going to look like. For example, some reported that they only understood aspects of the training when they finally conducted their first therapy session, whereas others found the information to be overwhelming without sufficient previous therapy experience. Most therapists suggested improvements, such as splitting the training over 2 days, increasing role-plays, practicing mental imagery techniques, and having video resources to refresh information.

I really enjoyed the training. The little consolidation training we did closer at the time, was really helpful because it was like more role play. So, seeing how you would actually use the app and having like visual example of that and how conversation could go even though we had the scripts, it was just easier to kind of see how it would be like in an actual session. That I found that really helpful.

Therapist ID: 7

#### Empowering Autonomy Through Accessible Digital Support

Most therapists reported that the IMAGINATOR 2.0 app was user-friendly and age-appropriate. The audios were the most frequently accessed app feature, while mood logging was also useful for young people. Therapists valued the app to have multiple components, but opinions varied around which component should be defined as the key one: guided imagery audios, the support section, or the goal section.

The participant... had quite a positive reaction to the app, and she used it between sessions as well. I think for her it was quite helpful because it reminded her to use imagery even outside of our sessions...I think she found the box audio quite helpful. Mood logging was helpful. She was able to see that she had more good days than she actually thought.

Therapist ID: 5

The audios were the most helpful for one of my participants. She didn't use the app much, but she found audios helpful. So, the safe bubble and the other ones she did explore them in her free time a few times. I wouldn't say it was regular, but she did listen to them.

Therapist ID: 6

Therapists noted that a few young people were excited to use the app. However, they reported that the young people felt a major challenge was technical difficulties, which prevented young people from engaging further with the app outside therapy sessions, and a few patients abandoned the app because of these. For example, the screen goes black, and young people need to uninstall and reinstall the app a couple of times. Few therapists believed that the app should have more personalization to address patients’ needs, as individual preferences could hinder engagement. For example, some patients found the voices or accents in the audios to be jarring, which led them to stop using them.

#### Adapting Technology to Complement Care Pathways

Most therapists highlighted that the app needs to be more actively integrated within therapy sessions, which may help participants associate the in-session use and independence practice, potentially enhancing engagement and use outside of sessions: this could be done by having one more dedicated face-to-face session to guide patients through the app and reinforce its use.

We should sit down and see things together because sometimes they don’t do it on their own or they have difficulties with tracking mood etc. My young person was excited to use the app. Even though their initial reaction was positive, exploring it on their own did not work.

Therapist ID: 3

I think it would be easier to start using the app or have things to do in the app from all the face-to-face sessions because it kind of consolidates it every week. So, if there's some activity or task to do on the first session, in the second session, and then in the third session, and having them recording it, kind of sets them up for when they're going off by themselves, and then we can consolidate it further when we're doing the phone calls. I think it would make it a lot easier for them to not forget the app and actually kind of integrate it more into the module. Whereas right now it kind of feels like two separate things.

Therapist ID: 7

A few therapists thought that using an incentive system could encourage patients to use the app more. For example, making the app visible and accessible on the patient’s phone was important; improving the prompts or notifications system, and for the therapist to record audios were other suggestions.

Thinking about just different avatars for themselves. So, people having their own kind of avatar that they could create, you know? As you would in a game with different codes and hair and all of that, that would be a fun option. Make it more personal to them...

Therapist ID: 6

## Discussion

### Summary of Findings

This study extends our previous proof-of-concept trial [33] of an imagery-based intervention in young people who SH. Our study shows that FIT is, in principle, acceptable and safe. FIT also has potential at reducing SH behaviors in young people via a blended digital therapy approach. Therapists reported that IMAGINATOR 2.0 overall had a positive impact on young people’s coping skills to manage SH. They assessed mental imagery as a helpful technique, although some young people may find it difficult. Therapists also valued the app as a useful tool but suggested various areas for improvement. These results should be treated with caution, given key limitations in our study, such as the small sample size and absence of a comparator intervention. Areas of uncertainty around acceptability also remain, as our results may have been biased by participants who disliked the intervention dropping out of the study. We also highlight feasibility challenges that need addressing in future studies.

### Feasibility of IMAGINATOR 2.0

We replicated the recruitment rate of our previous study [33], consistent with other blended digital [54] and SH [16,55] interventions, and consistent with therapists’ views that IMAGINATOR 2.0 fills a gap in available interventions to support the high presentation of young people who SH [56]. In line with our previous study [33] and other brief CBT interventions for SH, adherence to face-to-face therapy was satisfactory (70%) [57,58]. Instead, the attrition rate (48.3%) was higher than expected and higher than that reported for other fully digital interventions for SH, which showed lower dropout rates (15% and 28%, respectively) [16,17]. Most dropouts happened after the completion of the 3 face-to-face sessions, possibly suggesting that participants might have perceived phone call sessions as less beneficial or engaging compared to face-to-face interactions. Therapists emphasized how phone calls can be distracting for both participants and them, which is consistent with previous literature, suggesting a lack of control of the environment during phone calls [59]. A few dropouts occurred early after session 1, perhaps as the motivational interview selected those still in a contemplative phase of behavior change [60]. We also observed that a few participants dropped out prior to starting therapy. Importantly, long waiting times between enrollment and starting the therapy sessions (due to the lack of promptly available therapists in the service) might have led to a change in needs [61,62]. For example, some young people declined starting therapy as they had not self-harmed since the baseline assessment, whereas 2 adolescents experienced increased suicidal risk while waiting for therapy and were moved to a different team. As emphasized by our therapists, SH has to be the patient’s main concern. Previous literature suggests that ambivalence around reducing SH is often present while not necessarily overt [57], and this ambivalence could persist during long waiting periods or emerge after the first therapy session. Consideration of how motivation is better explored in screening suitable participants should be included in future studies. Objective markers of motivation to stop self-harming (eg, cognitive tasks) not relying on self-report may bypass the risk that young people start therapy because they feel that “they have to,” and lead to more personalized approaches [63].

High attrition could be explained by the fact that participants disengaging from therapy after a few sessions were also lost to outcome assessment: a common occurrence in psychological interventions’ research [64,65]. As in other studies, the overlap between attrition and poor treatment adherence hindered our efforts to understand the reasons behind dropouts. Over the study duration, we noted that consistency in the research team conducting assessments and keeping in touch with participants during the intervention with brief messages improved retention. A highly variable duration of the intervention (between 9.7 and 36 weeks), with some participants experiencing several weeks’ gap between sessions, may also have facilitated disengagement.

Finally, it is important to note that IMAGINATOR 2.0 was delivered by clinicians trained in low-intensity therapies rather than highly specialized psychologists (except one), which increases its feasibility in the context of low-resource availability in mental health services.

### Acceptability of IMAGINATOR 2.0

The absence of serious adverse events and only one case of SH deterioration suggests that IMAGINATOR 2.0 is a safe intervention for young people who SH but have a low-to-moderate risk profile at the point of enrollment. Both participants’ quantitative satisfaction ratings and therapists’ views indicate that our intervention was acceptable and well-received. Adherence rates also indicate good acceptability of the therapy sessions, while engagement dropped for follow-up calls, suggesting these could be reduced or made optional. Overall, the IMAGINATOR 2.0 app was reported to be reliable, intuitive to understand, and attractive to both by young people and therapists. Young people’s low ratings of apps’ stimulation were reflected in some therapists’ suggestions around adding gamification or further personalization. Overall, this supports the idea that an app can enhance control and autonomy over one’s own care, which appears key in learning to manage SH behaviors [66]. Integrating therapists’ suggestions in future app versions could improve this by sustaining engagement with the app.

Therapists strongly supported our blended digital approach to reduce SH in young people but recommended a more structured integration of the app in the therapy sessions. Importantly, the need for better integration was also shared by young people [34]. This possibly reflects an ongoing challenge in maximizing the potential of academic-led digital technology for therapeutic interventions, with apps failing to show superior efficacy to standard care so far [17].

### Mental Imagery

Therapists perceived IMAGINATOR 2.0 as impactful and mental imagery as a useful tool to target young people’s goals, although our study was not able to test a causal link between the two. Our findings of reduced emotional dysregulation scores, reduced craving to SH, and higher motivation to stop self-harming support that mental imagery could be a cognitive process able to regulate emotions and motivation away from maladaptive and toward adaptive behaviors [67]. However, in the absence of a control arm, these psychopathology improvements cannot be attributed to the intervention. In the context of growing evidence on the efficacy of targeting distressing involuntary imagery or harnessing the power of helpful imagery in anxiety and depression [68], further testing of IMAGINATOR 2.0 should be conducted. Importantly, therapists’ view of the intervention and imagery also matches young people’s subjective experience that IMAGINATOR 2.0 produces an emotional and behavioral change [34].

The mechanism by which FIT may reduce SH remains to be tested. It is possible that FIT’s personalized imagery plan simulating a way out of distress may work by diminishing the sense of entrapment, a recognized motivational factor in SH [69]. Alternatively, FIT could work by offsetting SH mental imagery [22]. Another advantage of our approach is that the content of functional imagery is highly personalized to each individual’s formulation of their idiosyncratic SH functions, from “reducing distress” to “feeling something” [70,71]. Importantly, this personalization reflects recent guidance to approach SH in a more individualized way [15]. Interestingly, therapists felt that key elements that made the intervention “work” were young people understanding their own SH formulation and improving goal-setting ability, in keeping with the importance of personalization and including positive values in interventions [72]. They also described that the most used feature of the app was mental imagery audios.

Importantly, therapists initially anticipated that mental imagery would be difficult to implement, especially in younger participants, as they would find abstract concepts difficult. This was true in reality with those younger than 14 years. In fact, research suggests that vividness in mental imagery relies on both long-term and working memory, which may not be fully developed before the age of 14 years [73,74]. This may explain why younger individuals struggled more with controlling and altering mental images consistent with their cognitive and emotional developmental stage [75]. However, mental imagery techniques have been found effective in participants as young as 14 years [76,77].

Therapists stressed the importance of regular rehearsal of the mental imagery plan via the app. A better integration between app and therapy may address this by specifically setting exercises that boost mental imagery’s motivational elements, and via repeated pre-experiencing of positive emotions and desired outcomes. This would ensure participants enhance their ability to manage distress via imagery before reaching a crisis point [78]. Finally, therapists suggested that more direct targeting of emotion regulation, per se, would improve IMAGINATOR 2.0. This suggestion reflects young people wanting to target underlying difficulties, for instance, emotional dysregulation, rather than caring about reducing SH [79]. A focus on emotion regulation is also supported by the recent evidence on the efficacy of ERITA, a web-based emotion regulation program, at reducing SH episodes [16]. On the other hand, other factors may contribute to SH, and emotional dysregulation only explains parts of SH’s persistence [80,81], favoring IMAGINATOR 2.0’s individualized approach.

### Limitations

The small sample and absence of a comparator arm limit the interpretation of our findings. In the absence of a control arm, a reduction in SH behavior could be secondary to passage of time, concurrent interventions such as medication, or nonspecific factors such as additional human contact provided by the study participation rather than the intervention content. The study was conducted in a single location, and mostly had participants identifying as females, which also limits generalizability. As most participants who stopped therapy early did not complete the outcome assessment, we have poor insight into barriers to engaging in IMAGINATOR 2.0. Other limitations include the absence of a specific adverse effects measure and the lack of a formal assessment of therapists’ adherence to the treatment protocol.

### Future Directions

Notwithstanding its limitations, this early evaluation combines the strength of both qualitative and quantitative methodology, a strong lived experience input, and a manualized treatment delivered by multiple staff across routine services. Future studies should now test the efficacy of IMAGINATOR 2.0 in a randomized controlled trial after considering design changes to improve feasibility and acceptability, such as higher monetary incentives and more continuous research staff contact to encourage participation in outcome assessments; therapists employed as trial staff to avoid delays in therapy start, an additional face-to-face session but less follow-up sessions, or follow-up video instead of phone calls to better maintain the therapeutic connection [82]. Another strength is the development and evaluation of IMAGINATOR 2.0 based on the epidemiology of SH (mean age of onset at 14 years old, and peak prevalence at 16-25 years old) rather than the traditional 18 years cutoff, in line with the new framework of youth mental health [83]. If future randomized controlled trial confirms that age is not associated with outcomes, this may ease the future implementation and access to IMAGINATOR 2.0, overcoming the limitation of services’ age constraints. However, certain adjustments by age should be first implemented in the IMAGINATOR 2.0 app, so that visual and engagement aspects are suitable for both young adolescents and young adults [34]. The intervention should also be tested in different community settings where young people initially seek help, such as schools, general practitioners, and nonstatutory services. As mental imagery–based techniques appear to be underused by children and adolescent therapists, in particular, it will be important to develop more training, including videos and interactive resources. The intervention manual should include more structured session-by-session guidance on when and how to facilitate use of the app.

It is also crucial to investigate IMAGINATOR 2.0’s mechanisms of action: for example, testing if the intervention works by reducing SH mental imagery, if outcomes are influenced by individual cognitive characteristics (impulsivity or compulsivity) [84,85], and what app components directly influence treatment response. This will enable refining and personalizing the app design. Finally, given its general focus on emotion regulation and behavior change strategies, IMAGINATOR 2.0 could be adapted as an early intervention targeting a variety of dysregulated behaviors.

### Conclusion

In summary, IMAGINATOR 2.0 is the first blended digital intervention developed with and for young people who experience SH behavior, in line with National Institute for Health and Care Excellence guidelines on the long-term management of SH [1]. Together with our previous findings [33], we provide initial evidence that IMAGINATOR 2.0 is safe and supported by clinicians to fill the current treatment gap for young people with SH in mental health services. Its brevity and low clinician input could make IMAGINATOR 2.0 highly scalable across a variety of health services, highlighting the potential impact of future efficacy testing. However, design adaptations are needed to conduct a successful randomized controlled trial.

## Data Availability

The datasets generated or analyzed during this study are available from the corresponding author on reasonable request.

## Appendix

Multimedia Appendix 1 Methods.

Multimedia Appendix 2 Focus Groups Topic Guide.

Multimedia Appendix 3 Results.

## Abbreviations

CBT: cognitive behavioral therapy
DSM-5: Diagnostic and Statistical Manual of Mental Disorders (Fifth Edition)
FIT: functional imagery training
SH: self-harm
