# Supplementary material for: Early Evaluation of IMAGINATOR 2.0 Intervention Targeting Self-Harm in Young People: Single-Arm Feasibility Trial
Source: JMIR Form Res. 2026 Jan 26;10:e79496. doi: 10.2196/79496 (PMC12887553; doi:10.2196/79496)
Supplement: Multimedia Appendix 1 [file formative_v10i1e79496_app1.docx]

**METHODS**

MEASURES

At baseline, suicidality was measured to assess risk using the [Columbia-Suicide Severity Rating Scale](http://ajp.psychiatryonline.org/article.aspx?articleid=181033)(C-SSRS) which is widely used interview for suicide risk assessment and measurement of suicidal ideation and behaviour. The *Lifetime/Recent* version was used at screening to gather recent history of suicidality including suicidal ideation and/or behaviour. The scale measures four constructs: severity of ideation on a 5-point ordinal scale (from 1=wish to be dead, to 5=suicidal intent with plan); intensity of ideation via 5 items (e.g. frequency, duration etc.), each rated on a 5-point ordinal scale; behaviour rated on a nominal scale (including actual, aborted, and interrupted attempts, preparatory behaviour and non-suicidal self-injurious behaviour); and lethality rated on a 6-point ordinal scale. This measure was additionally used at the start of each face-to-face FIT session.

Quantitative acceptability measures were:

- Client Satisfaction Questionnaire (CSQ): This is an 8-item version [36] designed to provide a sensitive and comprehensive assessment of patient satisfaction with the therapy received. This was only administered post-treatment and sum scores were calculated for each participant. Each item is rated on a 4-point Likert scale, where responses range from 1 (very dissatisfied) to 4 (very satisfied). The individual scores from each item are summed to produce a total score for each participant, with a possible range of 8 (indicating low satisfaction) to 32 (indicating high satisfaction). Higher scores on the CSQ reflect greater overall satisfaction with the therapy received.
- User Experience Questionnaire (UEQ): This is an acceptability measure of app use designed to assess perceptions of the app, with items rated on a 7-point Likert scale ranging from -3 to +3. A score of -3 reflects the poorest user experience, while a score of +3 represents the highest level of satisfaction. A score of 0 indicates a neutral experience. The questionnaire consists of 26 items divided across six subscales: Attractiveness (overall impression), Perspicuity (ease of understanding), Efficiency (task completion speed), Dependability (reliability), Stimulation (engagement and enjoyment), and Novelty (innovation and creativity). For each of the six dimensions, the mean score of the items that correspond to that dimension is calculated. Higher scores across these subscales reflect a better overall user experience, while lower scores highlight specific areas for improvement. For the purposes of this analysis, the average of all dimensions mean scores were calculated for each participant [37].

Secondary outcomes measures used in addition to measures of SH frequency and severity are listed below:

- Mental imagery related to self-harm: Using the *Self-Harm Imagery Interview* (SHII) adapted from [33,40]: This interview is about mental images that deal with self-harm or occur with self-harm. It asks participants to think about one of these and explores their thoughts and emotions in relation to the image. The PANAS+ measure specifically assesses positive emotions while the PANAS- assesses negative emotions on a 10-item scale each. Each item is rated on a 5-point Likert scale with responses ranging from 1 (not at all), very low intensity or absence of the specific emotion, to 5 (extremely), extremely high intensity of that emotion. Scores range from 10 to 50 (individually for PANAS+ and PANAS-), where a score of 10 reflects low positive/negative emotional intensity, indicating weak or minimal positive/negative emotions associated with the imagery, while a score of 50 represents high positive/negative emotional intensity, signifying strong and vivid positive/negative emotions. Higher scores indicate a greater intensity of positive/negative emotional experiences related to the imagery.
- Motivation to reduce self-harm: Using the State Motivation for Reducing Self-harm (SM-SH) scale [41]. This scale measures participants’ motivation to control their self-harm using 12 items that measure the strength of their motivational cognitions in the present moment. Each item is rated on a 10-point Likert scale, ranging from 0 (never), indicating that the participant has no motivation or desire to control their self-harm at that moment, to 10 (constantly), indicating that the participant has an extremely strong and persistent motivation to control their self-harm at that moment. Average scores are calculated for each participant ranging from 0 to 10, with higher scores indicating stronger motivation to control self-harm. This scale provides a sensitive measure of participants' current motivational state regarding self-harm reduction.
- Urge to self-harm: Using the *Craving Experience Questionnaire for Self-Harm* (CEQ-SH), adapted from CEQ [42]. This questionnaire assesses the urge to self-harm and assesses frequency, intensity, salience or dismissability of intrusive thoughts surrounding self-harm. It consists of 9 items, each rated on a 10-point Likert scale, ranging from 0 (not at all), indicating that the participant has no urge to self-harm or does not experience any intrusive thoughts related to self-harm, to 10 (constantly), indicating that the participant experiences a very strong and persistent urge to self-harm or is constantly preoccupied with thoughts of self-harm. The minimum score is 0, indicating no urge to self-harm, and the maximum score is 90, indicating the highest possible urge to self-harm. Higher scores reflect a stronger and more persistent urge to engage in self-harming behaviour.

*Mental health measures.*We selected what additional mental health measures to include together with the YPAG after discussion of what concurrent symptoms and behaviours they considered as relevant outcome of the intervention. These included low mood, anxiety, the ability to regulate emotions and other risky/maladaptive behaviours that are often concurrent to SH and may serve similar self-regulation purposes (e.g. substance misuse):

- Symptoms of low mood and anxiety: using 1) the *Revised Children’s Anxiety and Depression Scale (RCADS)* which is a 47-item measure for 12-17-year-olds with six sub-scales (separation anxiety disorder, social phobia, generalised anxiety disorder, panic disorder, obsessive compulsive disorder, and low mood (major depressive disorder)). The Anxiety sub-scales (37 items) consist of Separation Anxiety Disorder, Social Phobia, Generalised Anxiety Disorder, Panic Disorder, and obsessive-compulsive disorder. Each item is scored from 0 (never), indicating not experiencing the symptom described in the item during the past week, to 3 (always), consistently experiencing the symptom. Total scores range from 0 to 111. A total score is calculated by summing the individual item scores. Higher scores indicate more severe anxiety. In the Depression subscale (10 items), each item is scored from 0 (never), indicating not experiencing the symptom described in the item during the past week, to 3 (always), consistently experiencing the symptom. Total scores range from 0 to 30. A total score is calculated by summing the individual item scores. Higher scores indicate more severe depression. Here we report raw scores. The RCADS has been specifically developed for children and adolescents and has been validated in clinical and non-clinical samples [43, 87]. It has good internal consistency, test-retest reliability, and convergent validity. 2) the *Depression, Anxiety and Stress Scale (DASS-21)* [44] which is a 21-item scale for 18–25-year-olds measuring levels of depression, anxiety and stress over the past week. Each item is rated on a 4-point Likert scale, ranging from 0 (did not apply to me at all), indicating that the individual did not experience depression, anxiety, or stress symptoms during the past week to 3 (applied to me very much, or most of the time), indicating that the individual frequently experienced depression, anxiety, or stress symptoms. The Depression subscale assesses symptoms associated with dysphoric mood, such as feelings of worthlessness and consists of 7 items, with scores ranging from 0 to 21. The Anxiety subscale measures symptoms associated with physiological arousal (e.g., trembling) and includes 7 items, with scores ranging from 0 to 21. The Stress subscale measures symptoms of tension and reactivity to stressful events and includes 7 items, with scores for this subscale ranging from 0 to 21. Since the DASS-21 is a short version of the full scale, the total score for all subscales is calculated by summing the individual item scores and multiplying the final score by 2 to adjust for the reduced number of items. Higher scores on each subscale indicate more severe depression/anxiety/stress, reflecting a higher intensity of depressive symptoms, greater physiological arousal and anxiety symptoms, and greater tension and reactivity to stressors, respectively, in the past week. The DASS-21 has acceptable to excellent internal consistency validated in both clinical and non-clinical samples [88,89].
- Psychological wellbeing: using the Warwick-Edinburgh Mental Well-being Scale (WEMWBS) [45] which is a 14-item measure with positively worded items rated on a 5-point Likert scale, ranging from 1 to 5. A score of 1 ("none of the time") represents the lowest level of mental well-being, indicating that the individual rarely or never experiences the feelings or behaviours described in the item (e.g., feeling happy, confident, or connected). Conversely, a score of 5 ("all of the time") indicates the highest level of mental well-being, suggesting that the individual frequently or always experiences these positive mental health states. The total score is calculated by summing the individual item scores, with possible scores ranging from 14 to 70, where higher scores indicate better mental well-being and a greater level of positive mental health. The WEMWBS has shown good internal consistency and construct validity across adolescents and young adults aged 13 and over in both clinical and non-clinical populations [90-92].
- Emotion regulation abilities: using the *Difficulties in Emotion Regulation Scale-Short Form* [39] which is an 18-item scale adapted from the original long version that measures general emotion regulation ability as well as various aspects of emotion regulation (in six subscales), e.g. difficulties engaging in goal directed behaviour when upset, and limited access to emotion regulation strategies. Each item is rated on a 5-point Likert scale, with 1 representing "Almost Never" (0-10% of the time), indicating minimal difficulty with emotion regulation, and 5 representing "Almost Always" (91-100% of the time), indicating frequent or almost constant difficulty. The total score is calculated by summing the item scores. The scale has a minimum value of 18 and a maximum value of 90. Higher scores indicate greater emotion regulation difficulties, reflecting more significant challenges in managing and regulating emotions. Conversely, lower scores suggest better emotion regulation skills and fewer difficulties in handling emotional responses. The DERS has been validated in both adolescents and adults, demonstrating strong reliability and construct validity [39,93].
- Engagement in other risky, self-destructive behaviours (e.g., binge eating, substance misuse): using the *11-item behaviour supplement to the Borderline Symptom List* [46]. This is an 11-item scale measuring engagement in other risky, self-destructive behaviours (e.g., binge eating, substance misuse). The items are designed to assess the frequency and severity of these behaviours, which are commonly associated with borderline personality disorder. Each item is rated on a Likert scale from 0 to 4, with 0 representing "Not at all", indicating no engagement in the behaviour during the assessed period, and 4 representing "Daily or more often", indicating the participant engaged in the behaviour on a daily basis or more frequently. The total score is calculated by summing the responses. The scale has a minimum value of 0 and a maximum value of 44, with higher scores indicating more frequent or severe engagement in these behaviours. The scale has high internal validity and test-retest reliability in both adolescents and adults [94].
- Alcohol misuse/dependence: using the Alcohol Use Disorders Identification Test (AUDIT) [47]. This is a 10-item measure of alcohol misuse and possible dependence, in which participants are asked to rate items related to their drinking (e.g. 'How often do you have a drink containing alcohol') on a 5-point scale ranging from 0 to 4. A score of 0 indicates "Never" engaging in the behaviour (e.g., never drinking alcohol or never experiencing a specific consequence), while a score of 4 represents "Daily or almost daily", reflecting the highest level of engagement. The AUDIT is an effective means of identifying hazardous or harmful drinking behaviour. The total score is obtained by summing the individual item scores, with the scale ranging from 0 to 40. Higher scores indicate a greater risk of alcohol misuse, with scores of 8 or higher commonly used to identify individuals at risk for hazardous or harmful drinking. Conversely, lower scores suggest minimal or no alcohol-related problems. The AUDIT is validated for adults [95] and has been used reliably in adolescents [96].
- Cannabis misuse/dependence: using the Cannabis Use Disorder Identification Test Revised (CUDIT-R) [48]. This is an 8-item measure used to screen for problem cannabis use. Participants are asked to rate a series of items related to their cannabis use (e.g., "How often do you use cannabis") on a 5-point Likert scale, where 0 represents "Never" or no engagement in the behaviour, and 4 represents "Daily or almost daily", indicating the highest level of cannabis use. The total score is calculated by summing the responses, with the scale ranging from 0 to 40. A higher score indicates greater severity of cannabis misuse, and individuals with higher scores may be at risk for problematic cannabis use. Conversely, a lower score suggests minimal or no problematic cannabis use. The CUDIT is validated for both adults and adolescents with strong screening accuracy [97,98].

AGE CONSIDERATIONS

We selected outcome measures that were validated across adolescent and adult populations. When this was not possible, we included separate measures depending on age.

As mentioned in the main text, age was not associated with any feasibility outcomes. However, as shown in Figure 4, participants > 18 years old tended to present a higher number of self-harm episodes at baseline, which likely reflects differences in thresholds to access children vs adult secondary mental health services in the UK, with adult services being limited to more severe/engrained mental health presentations. Future larger studies are needed whether age moderates treatment outcomes. Pragmatically, we also noted that only two participants < 14 years old were referred to the study and enrolled, but did not complete the intervention. While this could be due to a multiplicity of reasons rather than just age, feedback from clinical teams involved in the study considers that for adolescents < 14 years old, parental involvement remains a key factor. We suggest that as an individual-based intervention future RCTs of Imaginator 2.0 should include young people aged 14-25. Future work should also explore how parents engagement could be included in the protocol, e.g. via psycho-education material, when appropriate and requested by young people.”

ANALYSIS

*Qualitative data*

In line with Braun and Clarke’s guidance on Reflexive Thematic Analysis (2015, 2021, 2022), we acknowledge that the research team’s positions shaped the interpretation of the focus group data. The team included a young research assistant (AS), three young people with lived experience of self-harm (SM, NC, AM), a trainee psychiatrist (EBG), an experienced qualitative researcher (LD), and a consultant psychiatrist specialising in self-harm (MDS). Lived-experience researchers contributed important sensitivity to the context of self-harm but also engaged in reflexive discussion to consider how personal experiences might influence interpretation. Clinical team members (EBG, MDS) brought expertise in therapeutic practice and feasibility assessment, while reflecting on how research interest in mental imagery (MDS) and general professional assumptions and experiences in mental health services could shape analytic decisions. Reflexive conversations were integrated throughout coding and theme development to ensure that these varied perspectives enriched, rather than constrained, the interpretation of therapist accounts.

### References

36. Coppersmith DDL, Bentley KH, Kleiman EM, Nock MK. Variability in the Functions of Nonsuicidal Self-Injury: Evidence From Three Real-Time Monitoring Studies. Behav Ther. 2021 Nov;52(6):1516-28.

37. Nock MK, Prinstein MJ, Sterba SK. Revealing the form and function of self-injurious thoughts and behaviors: A real-time ecological assessment study among adolescents and young adults. J Abnorm Psychol. 2009 Nov;118(4):816-27.

39. Baddeley AD, Andrade J. Working memory and the vividness of imagery. Journal of Experimental Psychology: General. 2000;129(1):126-45.

40. Kosslyn SM, Margolis JA, Barrett AM, Goldknopf EJ, Daly PF. Age Differences in Imagery Abilities. Child Development. 1990;61(4):995-1010.

41. Burnett Heyes S, Lau JYF, Holmes EA. Mental imagery, emotion and psychopathology across child and adolescent development. Developmental Cognitive Neuroscience. 2013 July 1;5:119-33.

42. Schwarz SM, Feike M, Stangier U. Mental Imagery and Social Pain in Adolescents—Analysis of Imagery Characteristics and Perspective—A Pilot Study. Children (Basel). 2021 Dec 8;8(12):1160.

43. Schwarz SM, Stangier U. Contents and Characteristics of Mental Imagery and their Association with Emotional Intensity in Adolescents: A Pilot Study. J Rat-Emo Cognitive-Behav Ther. 2023 Dec 1;41(4):838-55.

44. Sosic-Vasic Z, Schaitz C, Mayer B, Maier A, Connemann B, Kroener J. Treating emotion dysregulation in patients with borderline personality disorder using imagery rescripting: A two-session randomized controlled trial. Behaviour Research and Therapy. 2024 Feb 1;173:104454.

45. Knowles S, Sharma V, Fortune S, Wadman R, Churchill R, Hetrick S. Adapting a codesign process with young people to prioritize outcomes for a systematic review of interventions to prevent self-harm and suicide. Health Expectations. 2022;25(4):1393-404.

46. Edmondson AJ, Brennan CA, House AO. Non-suicidal reasons for self-harm: A systematic review of self-reported accounts. Journal of Affective Disorders. 2016 Feb 1;191:109-17.

47. Warne N, Heron J, Mars B, Solmi F, Biddle L, Gunnell D, et al. Emotional dysregulation in childhood and disordered eating and self-harm in adolescence: prospective associations and mediating pathways. Journal of Child Psychology and Psychiatry. 2023;64(5):797-806.

48. Chen PV, Helm A, Caloudas SG, Ecker A, Day G, Hogan J, et al. Evidence of Phone vs Video-Conferencing for Mental Health Treatments: A Review of the Literature. Curr Psychiatry Rep. 2022;24(10):529-39.

86. Donnelly A, Fitzgerald A, Shevlin M, Dooley B. Investigating the psychometric properties of the revised child anxiety and depression scale (RCADS) in a non-clinical sample of Irish adolescents. J Ment Health. 2019 Aug;28(4):345-56.

87. Park SH, Song YJC, Demetriou EA, Pepper KL, Thomas EE, Hickie IB, et al. Validation of the 21-item Depression, Anxiety, and Stress Scales (DASS-21) in individuals with autism spectrum disorder. Psychiatry Research. 2020 Sept 1;291:113300.

88 Gomez R, Summers M, Summers A, Wolf A, Summers J. Depression Anxiety Stress Scales-21: Measurement and Structural Invariance Across Ratings of Men and Women. Assessment. 2014 Aug 1;21(4):418-26.

89. Clarke A, Friede T, Putz R, Ashdown J, Martin S, Blake A, et al. Warwick-Edinburgh Mental Well-being Scale (WEMWBS): validated for teenage school students in England and Scotland. A mixed methods assessment. BMC Public Health. 2011 June 21;11:487.

90. McKay MT, Andretta JR. Evidence for the Psychometric Validity, Internal Consistency and Measurement Invariance of Warwick Edinburgh Mental Well-being Scale Scores in Scottish and Irish Adolescents. Psychiatry Research. 2017 Sept 1;255:382-6.

91. Trousselard M, Steiler D, Dutheil F, Claverie D, Canini F, Fenouillet F, et al. Validation of the Warwick-Edinburgh Mental Well-Being Scale (WEMWBS) in French psychiatric and general populations. Psychiatry Research. 2016 Nov 30;245:282-90.

92. Bhat NA, Roopesh BN, Bhaskarapillai B, Chokkanathan S, Benegal V. Difficulties in Emotion Regulation Scale-Short Form (DERS-SF): Psychometric Validation and Measurement Invariance Testing in a Sample of Urban Indian Adolescents. Indian J Psychol Med. 2025 Mar;47(2):119-26.

93. Goldbach RE, Neukel C, Panizza A, Reinken A, Krause-Utz A. Differentiating between intrapsychic symptoms and behavioral expressions of borderline personality disorder in relation to childhood emotional maltreatment and emotion dysregulation: an exploratory investigation. Eur J Psychotraumatol. 14(2):2263317.

94. Villarosa-Hurlocker MC, Schutts JW, Madson MB, Jordan HR, Whitley RB, Mohn RC. Screening for alcohol use disorders in college student drinkers with the AUDIT and the USAUDIT: a receiver operating characteristic curve analysis. Am J Drug Alcohol Abuse. 2020 Sept 2;46(5):531-45.

95. Reichenheim ME, Interlenghi GS, Ferreira MF, de Moraes CL. The Alcohol Use Disorders Identification Test (AUDIT) in Adolescents: Using a Model-Based Approach to Identify Patterns of Alcohol Misuse. Substance Use & Misuse. 2021 Nov 10;56(13):1915-22.

96. Palmer A, Adamson S, Schroder R, Wood L. Psychometric Performance of the Cannabis Use Disorders Identification Test- Revised (CUDIT-R) in an Youth Clinical Sample. Community Ment Health J. 2025 July 23

97. Coelho SG, Hendershot CS, Quilty LC, Wardell JD. Screening for cannabis use disorder among young adults: Sensitivity, specificity, and item-level performance of the Cannabis Use Disorders Identification Test - Revised. Addictive Behaviors. 2024 Jan 1;148:107859.
