# Supplementary material for: Early Evaluation of IMAGINATOR 2.0 Intervention Targeting Self-Harm in Young People: Single-Arm Feasibility Trial
Source: JMIR Form Res. 2026 Jan 26;10:e79496. doi: 10.2196/79496 (PMC12887553; doi:10.2196/79496)
Supplement: Multimedia Appendix 2 [file formative_v10i1e79496_app2.docx]

**FOCUS GROUP TOPIC GUIDE**

1. **Therapy**

- **General experience and expectations:** *How would you describe your overall experience with using the Imaginator intervention?*
- *What were your initial expectations and goals when using the Imaginator intervention?*
- *Why did you take part in the trial?*
- *Were there any particular concerns about using the Imaginator intervention? (cue to discuss concerns about the app)*
- **Clinical need:** *Can you tell us about what the clinical need/ patients need is for the Imaginator intervention in your service? (Did you feel the Imaginator intervention was needed / missing in your service /filling a gap or not? and if yes how?)*
- *What do you think of the selection of patients? Would you have selected them differently? Why? How? Who benefitted more or less from it?*
- *Could you make a vignette of the ‘ideal’ patient for Imaginator (eg for a triage meeting)?*
- **Structure of therapy:** *How did you find the structure of the therapy (3 f2f and 5 phone calls)? Was it enough (from your experience)? How was it for you and who for the patients? Why?*
- *Would you change anything about the structure of the therapy (length/hybrid style/number of sessions)? If so, what? Why?*
- *How did you find the follow-up phone calls? How was it for you and who for the patients? Would you change anything? Why?*
- **Therapy ‘mechanisms’:** *What do you think is the key component of the therapy? Why? (e.g. if you could only do one session what would it involve? (formulation, motivational interviewing, imagery plans, app?)*

1. **Mental Imagery**

- **Use of mental imagery:** *What did you think about the mental imagery technique you used in your therapy sessions?*
- *Can you share any success stories related to the use of mental imagery? Why do you think this is? In your opinion, what are the key strengths/advantages of mental imagery?*
- *Could you share specific examples of participants feedback on imagery? And if so, how you adapted mental imagery to individual client needs or feedback?*
- *Can you describe the most significant challenges and barriers you encountered when using mental imagery? Were there any unexpected or unintended consequences or outcomes of using mental imagery?) (Cue to discuss both patients’ difficulties and therapists’ difficulties with imagery)*
- *Would you use Mental Imagery (for the management of SH) in the future if given the opportunity to do so?*
- **Improvements / future use of mental imagery:** *What modifications or improvements do you think could enhance the effectiveness of mental imagery? Why?*
- *Did you collaborate with the other therapists while using mental imagery? If so, how did this collaboration impact the outcomes?*

1. **App**

- **App use by therapists:** *Can you tell us about your experience in using the app?*
- *What did you think about the app?*
- *Can you describe the main features and functions of the app you used in your therapy?*
- *Can you tell me more about how the app was introduced to you? And what do you think about it?*
- **Therapists experience of patients’ app use:** *How did you introduce and explain the app to your participants? How did they react to the idea of incorporating technology into their therapy?*
- *Can you share examples of specific cases where the app significantly contributed to the therapeutic process and outcomes?*
- *Were there any particular participant populations or issues for which you found the app to be especially effective? Or the opposite?*
- *What challenges or obstacles did you encounter when using the app in your therapy sessions, and how did you address them?*
- **App ‘mechanisms’:**
- *In your opinion what are the strengths/advantages of using this app in therapy? What about limitations/weaknesses?*
- *What do you think is the key component of the app? E.g. if you could only have one functionality, what would it be? (if they all say audios, what audios?)*
- **Future of the Imaginator App:** *Is there anything that you would change in the app? What more features could it have?*
- *What would you change in how app and therapy are integrated?*
- *What advice would you give other therapists considering the use of this app in their practice?*

1. **Training and future**

- **Training and supervision**: *How did the training prepare you for the actual therapy?*
- *Did you feel the therapy/course was adequately explained?*
- *Do you think anything else could be done that might have helped you with the therapy overall? If yes, what else do you think could be done more in training?*
- *Did you feel adequately supported during the sessions?*
- **Implementation:** *Would you adopt the Imaginator intervention in your service or not? Why?*
- *If you were to offer advice to other therapists considering implementing this intervention, what would it be?*
- *Are there any potential barriers to the adoption of the Imaginator intervention among therapists in the future? How can they be addressed?*
- *What do you think about integrating the app in particular into clinical care?*
- Is there anything we haven’t covered that you would like to share?
