# Supplementary material for: Early Evaluation of IMAGINATOR 2.0 Intervention Targeting Self-Harm in Young People: Single-Arm Feasibility Trial
Source: JMIR Form Res. 2026 Jan 26;10:e79496. doi: 10.2196/79496 (PMC12887553; doi:10.2196/79496)
Supplement: Multimedia Appendix 3 [file formative_v10i1e79496_app3.docx]

**RESULTS**

*Quantitative data*

Changes in clinical secondary outcomes are reported in Tables 2 and 3 showing a reduction in craving for SH and increase in participants’ motivation to reduce SH. Depression, anxiety, and stress also reduced in participants aged 18 or over, while wellbeing and emotion regulation skills scores increased.

Table S1: Change in self-harm related measures over 3-months after IMAGINATOR 2.0 (*n* = 15).

| Measure | Target | Baseline (T0) | Outcome Assessment (T1) | Median Difference | Effect size (r) |
| --- | --- | --- | --- | --- | --- |
|  |  | Median (IQR) | Median (IQR) |  |  |
| CEQ | Urge to SH | 59 (26.5) | 40 (31) | -13 (23) | 0.65 |
| SM-SH | Motivation to reduce SH | 5.33 (1.73) | 6.33 (2.12) | 1.42 (1.88) | 0.59 |
| SHII (PANAS+) | Mental imagery of SH – positive emotions | 14 (9) | 16 (5) | 1 (6) | 0.02 |
| SHII (PANAS-) | Mental imagery of SH – negative emotions | 33 (11.5) | 36 (10.5) | -2 (9.5) | 0.04 |

Legend. TLFB; Timeline Follow back Technique, CEQ; Craving Experience Questionnaire, SM-SH; State Motivation for Reducing Self-Harm, SHII; Self-Harm Imagery Interview, PANAS+; Positive and Negative Affect Schedule, positive items; PANAS-; Positive and Negative Affect Schedule, negative items.

Table S2: Change in mental health and wellbeing measures over 3-months after IMAGINATOR 2.0 (*n* = 15).

| Measure | Target | Baseline (T0) | Outcome Assessment (T1) | Median Difference | Effect size (r) |
| --- | --- | --- | --- | --- | --- |
|  |  | Median (IQR) | Median (IQR) |  |  |
| **RCADS** |  |  |  |  |  |
|  | Anxiety | 57 (29) | 49 (11.25) | -15 (15.5) | 0.60 |
|  | Depression | 18 (6) | 14.5 (5.75) | -2.5 (4) | 0.33 |
| **DASS-21** |  |  |  |  |  |
|  | Depression | 36 (4.5) | 34 (16.5) | -3 (13.5) | 0.69 |
|  | Anxiety | 36 (16) | 22 (8) | -6 (6.5) | 0.88 |
|  | Stress | 37 (5.5) | 28 (7) | -5 (12.5) | 0.67 |
| WEMWBS | Psychological wellbeing | 32 (11.5) | 41 (9.25) | 6 (11.5) | 0.66 |
| 11-items | Engagement in risky, self-destructive behaviours | 10 (4.5) | 10 (6.75) | -1 (3.5) | 0.28 |
| CUDIT | Cannabis misuse/dependence | 0 (1.5) | 0 (0) | 0 (1.5) | 0.44 |
| AUDIT | Alcohol misuse/dependence | 3 (11) | 1 (4) | 0 (6) | 0.04 |
| DERS-SF | Emotion regulation abilities | 66 (12.5) | 55.5 (12.25) | -7 (13.5) | 0.63 |

Legend. RCADS; Revised Children’s Anxiety and Depression Scale, DASS-21; Depression, Anxiety and Stress Scale, WEMWBS; Warwick-Edinburgh Mental Well-being Scale, 11-items; 11-item behaviour supplement to the Borderline Symptom List, CUDIT; Cannabis Use Disorder Identification Test Revised, AUDIT; Alcohol Use Disorders Identification Test, DERS-SF; Difficulties in Emotion Regulation Scale-Short Form.
